# P-186. Longitudinal Seropositivity of Antibodies Against Arboviruses Among an Agricultural Worker Cohort [AGRI] in Southwest Guatemala

**DOI:** 10.1093/ofid/ofaf695.409

**Published:** 2026-01-11

**Authors:** Julio del Cid-Villatoro, Neudy C Rojop, Daniel Vásquez, Cassandra Waltman, Claire Bradley, Blair Weikel, Diva M Barrientos, Chandler Bradley, Mattie Cassaday, Kareen Arias, Molly Lamb, Prem Lakshmanane, Rosemary Rochford, May Chu, Ross Kedl, Daniel Olson

**Affiliations:** Fundación para la Salud Integral de los Guatemaltecos, Retalhuleu, Retalhuleu, Guatemala; Fundacion Para La Salud Integral de los Guatemaltecos, Los Encuentros, Retalhuleu, Guatemala; Fundación para la Salud Integral de los Guatemaltecos, Retalhuleu, Retalhuleu, Guatemala; University of Colorado Anschutz, Aurora, Colorado; Fundación para la Salud Integral de los Guatemaltecos, Retalhuleu, Retalhuleu, Guatemala; University of Colorado Anschutz Medical Campus, Aurora, CO; Fundación para la Salud Integral de los Guatemaltecos, Retalhuleu, Retalhuleu, Guatemala; University of Colorado School of Medicine, Aurora, Colorado; University of Colorado Department of Immunology & Microbiology, Aurora, Colorado; Fundacion Para La Salud Integral de los guatemaltecos, Los Encuentros, Retalhuleu, Guatemala; Colorado School of Public Health, Aurora, Colorado; University of North Carolina, Chapel Hill, North Carolina; University of Colorado, Anschutz Medical Campus, Aurora, Colorado; University of Colorado Anschutz Medical Campus, Aurora, CO; University of Colorado Department of Immunology & Microbiology, Aurora, Colorado; CU School of Medicine, Denver, Colorado

## Abstract

**Background:**

Zika virus (ZIKV), dengue virus (DENV) and chikungunya virus (CHIKV) are arboviruses transmitted by Aedes mosquitoes. While DENV serotypes 1 and 2 (DENV1 and DENV2) are the most prevalent in Guatemala, serotypes 3 and 4 (DENV3 and DENV4) have caused recent epidemics. In 2024, a public health emergency was declared by the Ministry of Health due to the rise in dengue cases. CHIKV and ZIKV outbreaks were reported between 2014-2017.

**Figure:**
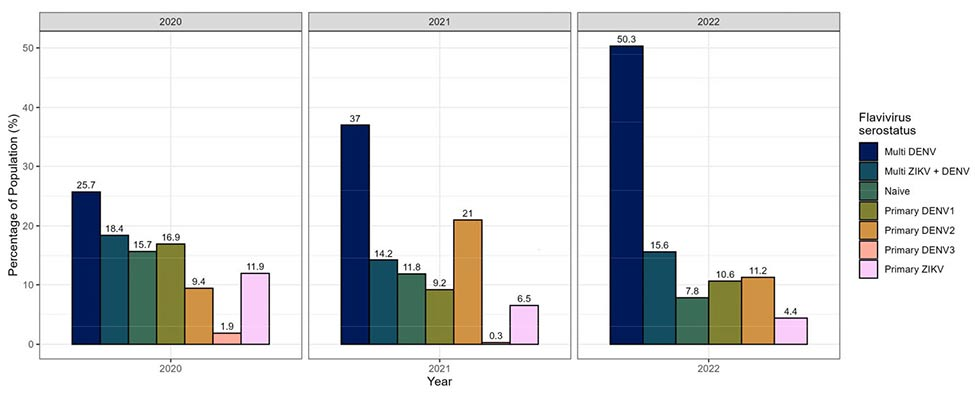
Evolution of flavivirus serostatus among the AGRI cohort (2020-2022)

The graph shows the serologic characteristics of the AGRI cohort against flavivirus infections. A rise in the multytipic DENV serostatus is observed over the years. On the contrary, there is a decline in the flavivirus-naive population.

**Figure:**
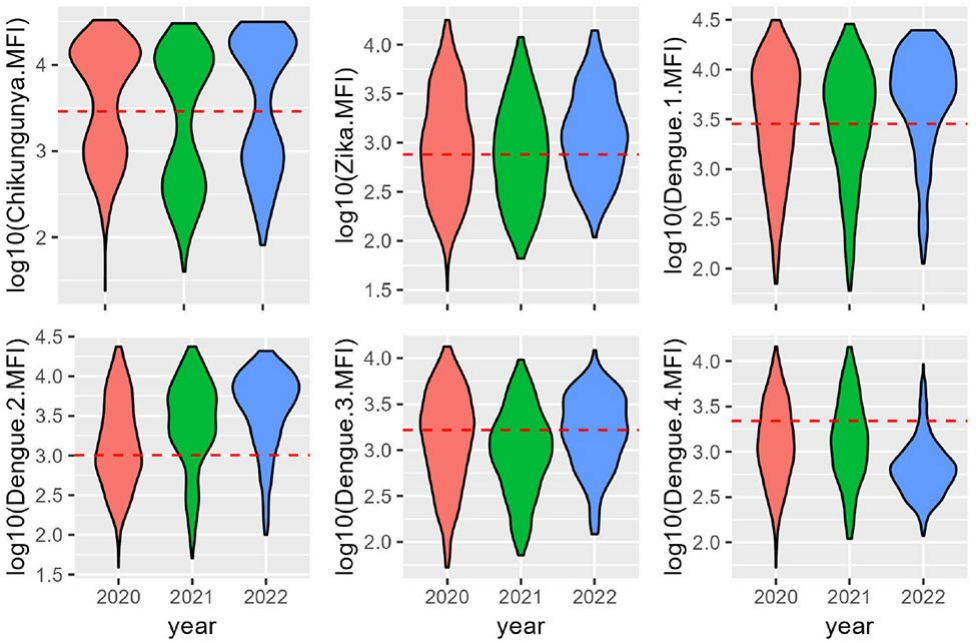
Density distribution of the MFI values against arbovirus-specific antigens

The MFI of the antigen-antibody reaction was recorded for each arbovirus. The evolution of cohort-level mean fluorescence intensity (MFI) readings over 3 years is shown. Positivity cutoffs are indicated by the dashed red line.

**Methods:**

Mean Fluorescence Intensity (MFI) from anti-CHIKV/ZIKV/DENV1-4 antibodies was obtained using an IgG-based multiplex bead assay. Flavivirus-specific beads were coupled with the EDIII protein of ZIKV/DENV1-4. CHIKV beads were coupled with CHIKV E2 protein. We tested 1136 samples from 478 individuals, collected in 2020 (n=478), 2021 (n=338) and 2022 (n=320). A set of 15 samples from children born between 2018-2022 were used as regionally appropriate samples to calculate the positivity cutoffs (mean MFI + 3SD). A previously described algorithm for analyzing flavivirus MFI signals was used to discriminate between naive samples and primary/multitypic infections.

**Results:**

CHIKV seropositivity was 56.9%, 51.5% and 53.4% for 2020, 2021 and 2022. During the follow up, 12 individuals had a negative-to-positive seroconversion against CHIKV. For 2020, 2021, and 2022, we detected seropositivity for primary infections of DENV1 (16.9%, 9.17%, 10.6%), DENV2 (9.41%, 21.0%, 11.2%), DENV3 (1.88%, 0.30%, 0.00%) and ZIKV (11.9%, 6.51%, 4.38%). Naïve-to-primary seroconversions were found for DENV1 in 2022 (n=12), for DENV2 in 2021 (n=14) and 2022 (n=10), and for ZIKV in 2021 (n=1) and 2022 (n=3). Flavivirus-naive prevalence went from 15.7% in 2020 to 7.8% in 2022.

**Conclusion:**

Though confirmatory testing is needed, these findings suggest that active DENV1/2, ZIKV and CHIKV transmission was ongoing in the region for this period.

**Disclosures:**

Blair Weikel, MPH, Merck: Grant/Research Support Molly Lamb, PhD, Merck: Grant/Research Support Daniel Olson, MD, Fundacion para la Salud Integral de los Guatemaltecos: Board Member|Merck: Grant/Research Support|Roche Diagnostics: Grant/Research Support

